# Use of emergency primary care among pregnant undocumented migrants over ten years: an observational study from Oslo, Norway

**DOI:** 10.1080/02813432.2023.2237074

**Published:** 2023-07-24

**Authors:** Frode Eick, Odd Martin Vallersnes, Heidi E. Fjeld, Ingvil Krarup Sørbye, Sven Eirik Ruud, Cecilie Dahl

**Affiliations:** a Department of Community Medicine and Global Health, Institute of Health and Society, University of Oslo, Oslo, Norway; b Department of General Practice, Institute of Health and Society, University of Oslo, Oslo, Norway; c Oslo Accident and Emergency Outpatient Clinic, Department of Emergency General Practice, City of Oslo Health Agency, Oslo, Norway; d Department of Obstetrics, Division of Obstetrics and Gynaecology, Oslo University Hospital, Oslo, Norway

**Keywords:** Migrants, pregnancy, prehospital emergency care, antenatal care, access to primary care

## Abstract

**Objective:**

To compare consultations with pregnant undocumented migrants at emergency primary health care to consultations with pregnant residents of Norway.

**Design:**

A cross-sectional study of consultations at several time points.

**Setting:**

The study was conducted at the Oslo Accident and Emergency Outpatient Clinic (OAEOC), the main emergency primary care service in Oslo, Norway.

**Subjects:**

Consultations with pregnant patients without a Norwegian identity number seeking care at the Department of Emergency General Practice at the OAEOC were identified through a manual search of registration lists from 2009 to 2019. The consultations were categorized by women’s residency status as ‘probably documented migrant’, ‘uncertain migrant status’, or ‘probably undocumented migrant’. We also extracted aggregated data for women with a Norwegian identity number (i.e. residents) presenting in consultations with pregnancy-related (ICPC-2 chapter W) conditions.

**Main outcome measures:**

Manchester Triage System urgency level at presentation, and hospitalization.

**Results:**

Among 829 consultations with female patients categorized as probably undocumented migrants, we found 27.1% (225/829) with pregnant women. About half of the pregnant women (54.6% (123/225)) presented with a pregnancy-related condition. Pregnant women that were probably undocumented migrants had an increased risk of being triaged with a high level of urgency at presentation (relative risk (RR) 1.86, 95% CI 1.14–3.04) and being hospitalized (RR 1.68, 95% CI 1.21–2.34), compared to pregnant residents.

**Conclusion:**

Pregnant undocumented migrants were more severely sick when presenting to emergency primary care services than pregnant residents. Increased access to primary care and emergency primary care services for pregnant undocumented migrants is urgently needed.

## Key points

Restricted access to primary care may increase the use of primary care facilities intended for emergency care.

A considerable proportion of the consultations with undocumented migrant women at the emergency primary care services are related to pregnancy.

Consultations with pregnant undocumented migrants more often contained severe pregnancy-related conditions compared to consultations with pregnant residents of Norway.

Interventions to increase access to primary care for pregnant undocumented migrants are urgently needed.

## 1. Introduction

Maternity care at the primary care level is essential to guide women in their pregnancy and to detect and treat adverse health conditions that may arise. In the Nordic countries, pregnant undocumented migrants have restricted access to primary care [1,2]. Since 2011 they have had the right to antenatal care in Norway but are still excluded from the regular general practitioner (RGP) scheme and reimbursement scheme, which restricts access to care. Restricted access may lead to suboptimal antenatal care, with an increased risk of adverse pregnancy outcomes [3–6]. Continuity of care, i.e. maintaining an RGP over time, is associated with fewer hospitalizations and less use of out-of-hours services [7]. Due to their precarious life situation, there is reason to believe that a trusting clinical relationship is especially important to pregnant undocumented migrants [8,9].

The regular general practitioner and reimbursement schemes are essential parts of the Norwegian health care system. As pregnant undocumented migrants do not have access to an RGP, they may have other health-seeking behaviours than residents, such as primarily using health facilities like midwives at Maternal and Child Health Centres (MCHC), non-governmental (NGO) clinics, or emergency primary care services [10–12]. Some undocumented migrants can access general practitioners, even if they do not have the rights [13]. However, in addition to restrictions in access to care, fear of deportation, financial difficulties, and lack of knowledge about rights and where to seek help may prevent them from getting timely care [9,14]. Previous reviews from Europe have shown that undocumented migrants underutilize both primary health care services, and antenatal primary health care specifically [15,16].

There might be similarities between the use of emergency primary care services among undocumented and documented migrants. Overall, documented migrants in Norway use emergency primary care services less than Norwegian-born residents [17]. Some subgroups have increased use of emergency care, which may be seen in relation to the use of other primary care services [18–21]. Studies suggest that subgroups of documented migrants may be overrepresented in emergency primary care due to lower affiliation with, or problems with accessing the RGP scheme [22,23]. However, we also know that legal status matters, as healthcare use is lower among undocumented compared to documented migrants in southern Europe [24,25].

The extent of, along with trends in, the use of emergency primary care services among undocumented migrants in the Nordic countries is an under-researched topic. Little is known about the use of emergency primary care services by pregnant undocumented migrants in Norway before and after the policy change in 2011 that granted them the right to antenatal care. As the total number of undocumented migrants in a society is both unregistered and in flux, the proportion of this population seeking care is difficult to measure over time. We therefore wanted to explore the trends in the use of emergency primary care service in Oslo among pregnant undocumented migrants and estimate the severity of their pregnancy-related condition at presentation. We hypothesized that pregnant undocumented women had a higher level of urgency at presentation and a higher risk of hospitalization than residents of Norway.

## 2. Material and methods

### 2.1. Design

Due to the lack of Norwegian identity numbers, undocumented migrants cannot be tracked over time. Hence, we collected and analysed cross-sectional samples of consultations from the following periods: 2009–2010, 2012–2013, 2015–2016, and 2018–2019. Years in between were left out due to the high workload of collecting data.

### 2.2. Setting

Oslo is the capital of Norway with a population of 699,827 as of 01.01.2022, according to Statistics Norway. The Norwegian healthcare system is two-tiered with a gate-keeping function, and patients must see a primary care doctor for referral to a hospital or be triaged for hospital care by the ambulance service. Residents are provided with a Norwegian identity number and have the right to an RGP. Antenatal care is provided by RGPs, who are mostly self-employed, and by midwives at municipal MCHC. The Oslo Accident and Emergency Outpatient Clinic (OAOEC) is the main emergency primary care service in Oslo and is open at all hours for everyone with an urgent medical need. The OAEOC comprises an emergency general practice service and an emergency social service run by the municipality, as well as a trauma clinic and an emergency psychiatric service run by Oslo University Hospital. When patients present at OAEOC, the urgency level is assessed, and a Manchester Triage System code is set by the reception nurse. The urgency level code may be adjusted while the patient is waiting to be seen by a doctor. Referral to hospitalization from the OAEOC is decided by the doctor treating the patient.

### 2.3. Participants and categorization of residency status

**Figure 1.**
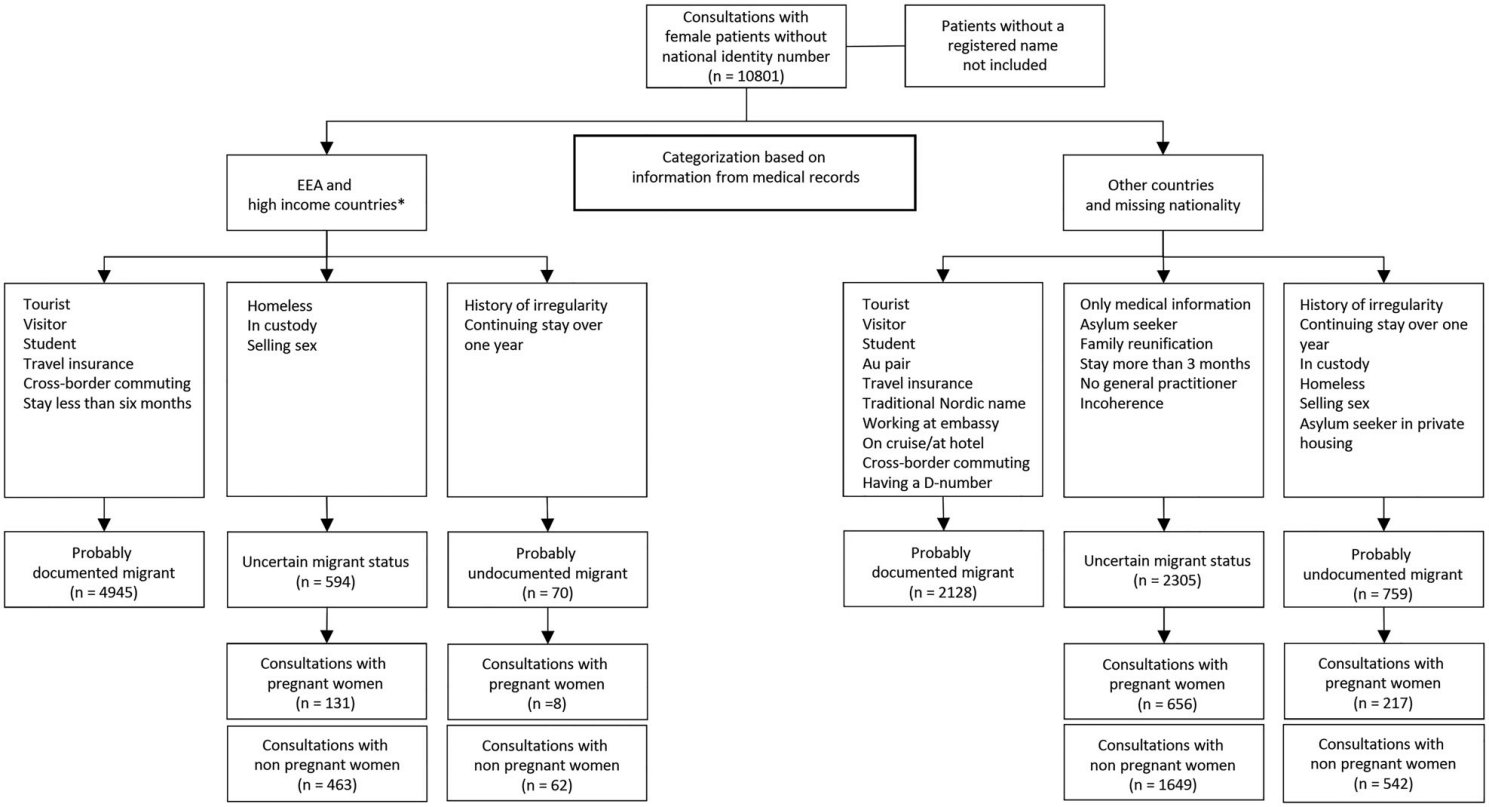
Female patient consultations at the Department of Emergency General Practice in Oslo by categorization of residency status 2009–2019**. *Switzerland, United Kingdom, United States of America, Canada, Australia, Singapore, Malaysia, Japan, South Korea, Saudi Arabia, Israel, United Arab Emirates, Qatar, Bahrain, Oman, and Kuwait. **Years not included: 2011, 2014 and 2017.

We searched the patient registration lists at the Department of Emergency General Practice (DEGP) at the OAEOC and included consultations with female patients without a Norwegian identity number from the eight inclusion years. For 2019 we also included consultations with male patients to estimate the female/male ratio of consultation. Consultations with patients without a registered name were not included as these patients most likely were residents of Norway unable to present a name due to intoxication. Consultations with patients without a Norwegian identity number were categorized by residence status as ‘probably documented migrant’, ‘uncertain migrant status’ (UMS), or ‘probably undocumented migrant’ (PUM), based on the information in the medical records. A flow chart for the categorization of female patient consultations is shown in Figure 1.

For comparison, we extracted aggregated data from women with a Norwegian identity number who had a consultation with an International Classification of Primary Care, 2nd version (ICPC-2) chapter W (Pregnancy, Childbearing, Family Planning) diagnosis from the same eight years. These women were assumed to be pregnant residents of Norway.

### 2.4. Data collection

From the included medical consultation records of patients without a Norwegian identity number, we recorded the patient’s sex, age, month and year of consultation, nationality, insurance, civil status, and whether the patient was pregnant.

From consultations with pregnant women with residence status UMS and PUM we registered Manchester Triage System code, hospitalization, documented use of a professional translator, and ICPC-2 main diagnosis in chapter W.

From consultations with pregnant residents of Norway (with a Norwegian identity number), we extracted aggregated data on the Manchester Triage System code, hospitalization, and mean age. We used the last updated Manchester Triage System code for each consultation.

### 2.5. Outcome measures

Our main outcome measures were the severity of the pregnancy-related condition (ICPC-2 chapter W diagnosis), measured as the level of urgency at presentation, and transferral to hospital. Manchester Triage System (five levels) code ‘1’ (Immediate) and ‘2’ (Very urgent) were set as high level of urgency at the presentation. In 2009 and 2010 the Manchester Triage System was not yet in use at the OAEOC. The locally developed triage system in use at the time had four levels, and codes ‘1’ and ‘*2’ in the local system were considered equivalent to codes ‘1’ and ‘2’ in the Manchester Triage System and set as high level of urgency.

### 2.6. Statistical analyses

Statistical analyses were performed using StataSE version 16. Descriptive characteristics of consultations with pregnant women by residency status, and factors describing their use of the OAOEC were summarized as number (n) and percentage (%), in total and over time. The outcome variables, high level of urgency and hospitalization, were found to be over-dispersed. To compare the severity of pregnancy-related conditions in consultations with PUM and women with UMS to consultations with pregnant residents of Norway, we, therefore, used negative binominal regression to estimate Relative Risk (RR) with 95% confidence intervals (95% CI). A robust variance estimator was applied to account for the potential clustering of consultations by patients. Directed Acyclic Graphs were used to illustrate the relation between exposure (resident status), covariates, and outcomes, and to determine which potential confounders to include in the multivariable regression. We adjusted for age as pregnant migrants are often younger than pregnant residents of Norway. The significance level was set to 0.05.

### 2.7. Ethics

The study was approved by the Regional Ethical Committee 19.02.20 (REK Sør-Øst, case number 68329) with exemption from consent to obtain information. A Data Protection Impact Assessment (DPIA) was performed together with Norwegian Centre for Research Data and approved by the University of Oslo and the data protection officer of the City of Oslo.

## 3. Results

### 3.1. Demographic characteristics

Women (pregnant and non-pregnant) with a consultation at the DEGP in the eight years studied who did not have a Norwegian identity number, originated from 145 different countries. Among 829 consultations with female patients categorized as PUM, we found 27.1% (225/829) consultations with pregnant women (Supplementary Table 1). About half of the consultations with pregnant women (54.6% (123/225)) and 14.8% (123/829) of all consultations with PUM were due to a pregnancy-related condition.

Table 1 shows the characteristics of consultations with pregnant women categorized as PUM and UMS. They originated from 78 different countries. Pregnant women categorized as PUM with the highest share of consultations were from Nigeria 15.1% (34/225), Somalia 14.2% (32/225) and Iraq 6.2% (14/225). Pregnant women categorized as UMS with the highest share of consultations were from Romania 12.9% (102/787), Syria 9.5% (75/787), and Somalia 7.1% (56/787). Romanians were mainly living on the street or at emergency shelters, and Syrians and Somalis were mainly asylum seekers living at asylum reception centres. Pregnant women categorized as PUM had a mean age of 27.8 (SD 4.9), women categorized as UMS 26.6 (SD 5.4), and residents of Norway 30.2.^1^

**Table 1.** Characteristics of pregnant women’s consultations without a Norwegian identity number presenting at an emergency primary care service in Oslo, Norway.

	Probably undocumented migrant		Uncertain migrant status	
	*n* = 225	%	*n* = 787	%
Maternal age				
Median (years)	28 (IQR 24–31)		26 (IQR 23–30)	
Missing information	0		5	
Self-reported region of origin				
EEA^a^	8	3.6	127	16.1
Europe & Central Asia	24	10.7	92	11.7
Middle East & North Africa	34	15.1	174	22.1
Sub-Saharan Africa	105	46.7	166	21.1
North America	0	0	1	0.1
Latin America & Caribbean	0	0	14	1.8
East Asia & Pacific	17	7.6	19	2.4
South Asia	10	4.4	34	4.3
Missing information	27	12.0	160	20.3
Diagnosis ICPC-2^b^				
W03 Antepartum bleeding	39	17.3	110	14.0
W29 Pregnancy symptoms other	27	12.0	68	8.6
W05 Pregnancy vomiting/nausea	14	6.2	65	8.3
W82 Abortion spontaneous	7	3.1	44	5.6
W90/92 Livebirth	1	0.4	1	0.1
W93 Stillbirth	0	0.0	1	0.1
Other W	35	15.6	148	18.8
Other than W	93	41.3	323	41.0
Missing	9	4.0	27	3.4
Manchester Triage System code				
1 (Immediate)	3	1.4	15	1.9
2 (Very Urgent)	47	20.9	172	21.9
3 (Urgent)	135	60.0	471	59.9
4 (Standard)	38	16.9	127	16.1
5 (Non-urgent)	1	0.4	1	0.1
Missing information	1	0.4	1	0.1
Transferred to hospital	66	29.3	211	26.8
Missing information	6	2.7	13	1.7
Documented use of a translator				
Yes	19	8.4	162	20.6
No	200	88.9	613	77.9
Missing information	6	2.7	12	1.5

^a^European Economic Area.

^b^W is the pregnancy chapter in ICPC-2.

### 3.2. Trends in use

**Figure 2.**
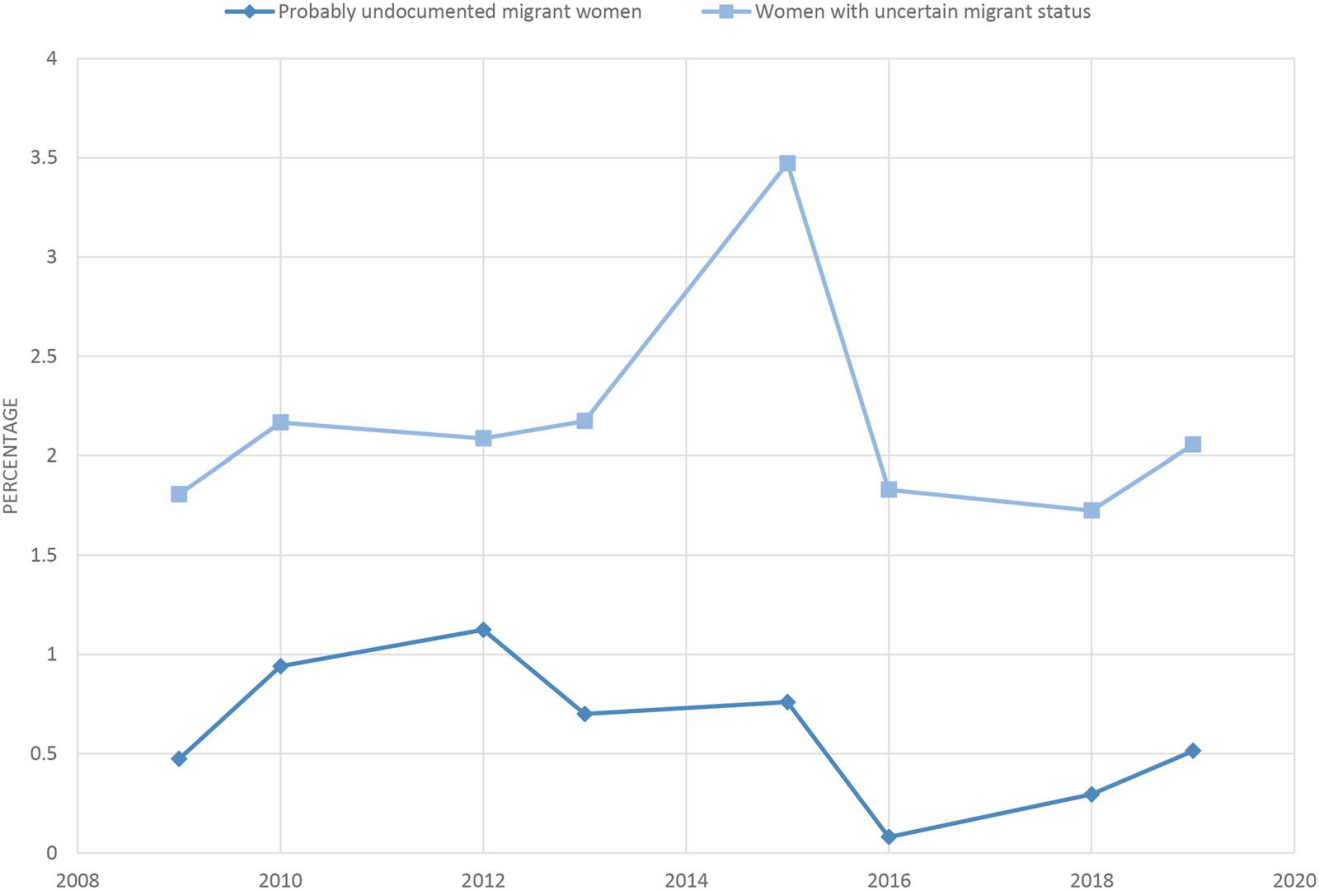
Proportions of total pregnancy-related consultations* at emergency primary care service among women with different residency status. *With ICPC-2 chapter W diagnosis.

The trends in number of consultations with pregnant women followed the trends of non-pregnant women categorized as PUM or UMS. The consultations for pregnant women categorized as PUM peaked in 2012 with 49 consultations, and for pregnant women categorized as UMS in 2015 with 178 consultations. In total, we found 28.1 consultations per year with pregnant women categorized as PUM, 98.4 consultations per year with those categorized as UMS and 2426 consultations per year with pregnant resident women. The consultations with women categorized as PUM or UMS were evenly distributed across the seasons. Less than 4% of the consultations with women presenting with a pregnancy-related condition at the emergency primary care service per year were with women categorized as PUM or UMS (Figure 2).

### 3.3. Reasons for presentation

The most frequent pregnancy-related conditions in consultations with both PUM and UMS were ‘W03 Antepartum bleeding’, ‘W29 pregnancy symptoms other’ and ‘W05 pregnancy vomiting/nausea’ (Table 1). Of the non-pregnancy-related conditions (ICPC-2 other than Chapter W) stomach pain and urinary tract infection were among the common health problems.

### 3.4. Severity of condition

Table 2 shows the urgency level at presentation in consultations among the different groups. Consultations with pregnant women categorized as PUM had an increased risk of being triaged with a high level of urgency at presentation compared to consultations with pregnant residents (RR 1.86, 95% CI 1.14 − 3.04). They also had an increased risk of containing information on women being hospitalized compared to consultations with pregnant residents (RR 1.68, 95% CI 1.21–2.34).

**Table 2.** Proportions and risk ratio of severe pregnancy-related conditions at presentation to an emergency primary care service in Oslo.

			Resident women		Women with uncertain migration status		Probably undocumented migrants		Women with uncertain migration status vs. resident women (ref.)		Probably undocumented migrants vs. resident women (ref.)	
	n	%	n	%	n	%	n	%	RR ^a^	95% CI	RR ^a^	95% CI
Total consultations	19968	100	19408	100	437	100	123	100				
High level of urgency at presentation	2168	10.9	2038	10.5	104	23.8	26	21.1	1.99	(1.13, 3.53)*	1.86	(1.14, 3.04)*
Hospitalized	3755	19.3	3581	18.5	136	31.1	38	30.9	1.62	(1.19, 2.22)**	1.68	(1.21, 2.34)**

^a^Adjusted for age.

*p-value < 0.05.

**p-value < 0.01.

CI: confidence interval; RR: risk ratio.

In consultations with pregnant women categorized as PUM, there was a similar risk of being triaged with a high level of urgency at presentation 21.1% (26/123) and hospitalized 30.9% (38/123) with pregnancy-related conditions (ICPC-2 chapter W) compared to non-pregnancy-related conditions (ICPC-2 other than chapter W): 25.8% (24/93) and 30.1% (28/93), respectively.

Consultations with pregnant women categorized as UMS also had an increased risk of being triaged with a high level of urgency at presentation (RR 1.99, 95% CI 1.13–3.53) and containing information on women being hospitalized (RR 1.62, 95% CI 1.19–2.22), compared to consultations with pregnant residents.

## 4. Discussion

### 4.1. Principal findings

In more than one in four (27.1%) of the consultations with women categorized as PUM, the patient was pregnant. Consultations with pregnant women categorized as PUM had an increased risk of being triaged with a high level of urgency at presentation to emergency primary care, and containing information on women being hospitalized, compared to consultations with pregnant residents of Norway.

### 4.2. Strengths and limitations

Studies considering the undocumented migrant population’s use of emergency primary care services are rare. We used pre-defined criteria for categorization by residency status that was applied diligently throughout the data collection process. Data collection and categorization were done by one researcher. We also had data from several years, which made it possible to observe trends in use. A previous study has found that the assigned diagnosis codes corresponded well with the medical record information [26]. However, we only had data on consultations and not at the patient level, and there may be misclassification in the categorization of consultations due to a lack of information in the patients’ medical records. We cannot rule out that pregnant women had several consultations at the DEGP, but we were not able to find any pregnant patients without a Norwegian identity number with several consultations according to their names. We assumed that pregnant residents of Norway only had one consultation due to their access to follow-up at RGPs, MCHCs and hospitals, which is why we used robust variance estimation. Consultations with a high level of urgency at presentation may have less personal information recorded, hence these consultations could end up in the category UMS, which is why we also included results for this group. Information on the patients’ length of stay in Norway and marital status, which could have been important explaining factors, or possible confounders, were generally not registered in the medical records. However, length of stay has not been identified as an important explanatory factor for the use of emergency primary care services among documented migrants [17].

### 4.3. Findings in relation to other groups

#### 4.3.1. Comparison to documented migrants

In the current study, there was a higher proportion of urgent consultations among pregnant women categorized as PUM compared to pregnant residents. This finding was contrary to our pre-defined hypothesis based on previous Nordic studies that have found a higher proportion of *non*-urgent consultations among *documented* migrants compared with other residents [22,27]. The reasons discussed were that some documented migrants, even with equal rights to healthcare, have reduced access to and/or lower affiliation with an RGP and therefore more often use emergency services for the treatment of non-urgent conditions [21,23]. Pregnant undocumented migrants in Norway do not have access to the RGP scheme, it was therefore expected that the same pattern would be found in this patient group. However, as opposed to documented migrants (who may also struggle with language barriers and how to navigate the welfare system), undocumented migrants have restricted rights, may fear deportation when approaching health care services, and have greater financial difficulties [9,11,14]. All these factors may contribute to pregnant undocumented migrants being more hesitant to use emergency services, even with restricted access to primary care.

#### 4.3.2. Comparison to resident women

One of the health-seeking alternatives previously identified in undocumented migrants is simply to do nothing [1,12]. Still, problems in pregnancy may coerce women to seek care. In the current study, more than one in four (27.1%) of the women categorized as PUM were pregnant, and 14.8% were given an ICPC-2 chapter W diagnosis. For comparison, the proportion given an ICPC-2 chapter W diagnosis among resident women seeking emergency primary care services in 2019 was only 1.3% [28]. This might imply that pregnant undocumented migrants are using emergency primary care services more than non-pregnant undocumented migrants, or that a larger proportion of undocumented migrant women are pregnant and in need of emergency primary care compared to resident women.

#### 4.3.3. Use of antenatal care in undocumented pregnant women

Studies from countries with supposedly universal access to primary care still report low use of antenatal care among undocumented migrant women. Undocumented migrant women in the Netherlands were found to have low use of primary care, and at the same time, unmet health care needs [29]. Pregnant undocumented migrants in the Netherlands also have an increased risk of inadequate antenatal care compared to documented migrants [30]. In France, having an undocumented status was associated with an increased risk of inadequate use of antenatal care compared to French-born women (OR 2.58 (95% CI 2.16–3.07)), and the risk was higher than in documented migrants [25]. This may indicate that increasing the use of antenatal care among pregnant undocumented migrants is more complex than simply granting them access to care.

The findings of low use of antenatal care in pregnant undocumented migrants are also supported by systematic reviews showing low use of both antenatal and other primary care among undocumented migrants, and lower use than residents [15,16]. Importantly, delay in seeking care has been found to be an underlying cause of the majority of maternal deaths among foreign-born women in Sweden [4]. Therefore, our finding of increased risk of a high level of urgency at presentation and hospitalization is likely to be caused by delay in seeking care.

#### 4.3.4. Possible contributing factors

Pregnant undocumented migrants’ vulnerable situation and unclear legislation may affect clinical decision-making [1,31]. Non-urgent consultations could be a selection of who uses the OAEOC. However, there are few other possibilities for emergency primary care services in Oslo than the OAEOC (mostly private emergency clinics that demand high consultation fees). The exception is one NGO clinic in Oslo, which is free of charge but was open only 1–3 days a week during the study period. In a previous study, we explored pregnant undocumented migrants’ use of this clinic and found 46.7 pregnancies per year in 2009–2020, compared to 28.1 in the current study [5]. The use of DEGP among pregnant undocumented migrants declined since 2012, concurrent with the increase in the use of the NGO clinic. New regulations in 2011 may also have contributed to this decline.

### 5. Conclusion and implications

In the current study, emergency primary care consultations with pregnant women categorized as probably undocumented migrants more often contained severe pregnancy-related conditions, and there was a higher risk of the women being hospitalized compared to consultations with pregnant residents. Adverse pregnancy-related conditions may increase in severity if left untreated, which implies that pregnant undocumented migrants are delaying seeking care. Clinicians in contact with pregnant undocumented migrants should be aware of their presently restricted access to primary care and strive towards equity in antenatal care. The development of measures to increase access to emergency primary care and other primary care services for pregnant undocumented migrants in line with resident women is urgently needed.

## Supplementary Material

Supplemental Material

Supplemental Material

## Data Availability

Due to the sensitivity, the data are available on reasonable request to the corresponding author.

## Note

1 Mean maternal age at childbirth in Norway in 2019: 30.3 (SD 4.8) Medical Birth Registry, read 02.12.2022.
