# Supplementary material for: Use of emergency primary care among pregnant undocumented migrants over ten years: an observational study from Oslo, Norway
Source: Scand J Prim Health Care. 2023 Jul 24;41(3):317–25. doi: 10.1080/02813432.2023.2237074 (PMC10478594; doi:10.1080/02813432.2023.2237074)
Supplement: Supplemental Material [file IPRI_A_2237074_SM3337.docx]

|  |  | Probably undocumented | | Uncertain  migration status | | Probably  documented | |
| --- | --- | --- | --- | --- | --- | --- | --- |
| Women ^[[1]](#endnote-1)^ | | n = 829 | % | n = 2899 | % | n = 7073 | % |
| Age (years) | |  |  |  |  |  |  |
|  | 18-30 | 452 | 54.5 | 1508 | 52.0 | 3104 | 43.9 |
|  | 31-40 | 193 | 23.3 | 706 | 24.4 | 1181 | 16.7 |
|  | 41-50 | 92 | 11.1 | 296 | 10.2 | 694 | 9.8 |
|  | 51-60 | 44 | 5.3 | 189 | 6.5 | 829 | 11.7 |
|  | >60 | 44 | 5.3 | 179 | 6.2 | 1258 | 17.8 |
|  | Missing information | 4 | 0.5 | 21 | 0.7 | 7 | 0.1 |
|  |  |  |  |  |  |  |  |
| Self-reported region of origin | |  |  |  |  |  |  |
|  | EEA ^[[2]](#endnote-2)^ | 67 | 8.1 | 580 | 20.0 | 4034 | 57.0 |
|  | Europe & Central Asia | 73 | 8.8 | 291 | 10.0 | 317 | 4.5 |
|  | Middle East &North Africa | 164 | 19.8 | 463 | 16.0 | 418 | 5.9 |
|  | Sub-Saharan Africa | 318 | 38.4 | 455 | 15.7 | 186 | 2.6 |
|  | North America | 3 | 0.4 | 7 | 0.3 | 592 | 8.4 |
|  | Latin America & Caribbean | 34 | 4.1 | 88 | 3.0 | 239 | 3.4 |
|  | East Asia & Pacific | 56 | 6.8 | 110 | 3.8 | 508 | 7.2 |
|  | South Asia | 38 | 4.6 | 139 | 4.8 | 113 | 1.6 |
|  | Missing information | 76 | 9.2 | 766 | 26.4 | 666 | 9.4 |
|  |  |  |  |  |  |  |  |
|  | Number of consultations  with pregnant women | 225 | 27.1 | 787 | 27.1 |  |  |
|  |  |  |  |  |  |  |  |
| Men ^[[3]](#endnote-3)^ | | n = 115 | % | n = 237 | % | n = 752 | % |
| Age (years) | |  |  |  |  |  |  |
|  | 18-30 | 28 | 24.3 | 63 | 26.6 | 224 | 29.8 |
|  | 31-40 | 28 | 24.3 | 79 | 33.3 | 179 | 23.8 |
|  | 41-50 | 37 | 32.2 | 39 | 16.5 | 113 | 15.0 |
|  | 51-60 | 17 | 14.8 | 33 | 13.9 | 82 | 10.9 |
|  | >60 | 1 | 0.9 | 22 | 9.3 | 151 | 20.1 |
|  | Missing information | 4 | 3.5 | 1 | 0.4 | 3 | 0.4 |
|  |  |  |  |  |  |  |  |
| Self-reported region of origin | |  |  |  |  |  |  |
|  | EEA ^b^ | 48 | 41.7 | 126 | 53.2 | 497 | 63.7 |
|  | Europe & Central Asia | 7 | 6.1 | 22 | 9.3 | 24 | 3.2 |
|  | Middle East &North Africa | 10 | 8.7 | 12 | 5.1 | 24 | 3.2 |
|  | Sub-Saharan Africa | 18 | 15.6 | 18 | 7.6 | 9 | 1.2 |
|  | North America | 2 | 1.7 | 0 | 0 | 65 | 8.6 |
|  | Latin America & Caribbean | 2 | 1.7 | 5 | 2.1 | 17 | 2.3 |
|  | East Asia & Pacific | 1 | 0.9 | 4 | 1.7 | 66 | 8.8 |
|  | South Asia | 2 | 1.7 | 1 | 0.4 | 11 | 1.5 |
|  | Missing information | 25 | 21.7 | 49 | 20.7 | 57 | 7.6 |

1. In eight years: 2009, 2010, 2012, 2013, 2015, 2016, 2018, 2019 [↑](#endnote-ref-1)
2. European Economic Area including Switzerland [↑](#endnote-ref-2)
3. In one year: 2019 [↑](#endnote-ref-3)
