# Supplementary figures and images for: Use of emergency primary care among pregnant undocumented migrants over ten years: an observational study from Oslo, Norway
Source: Scand J Prim Health Care. 2023 Jul 24;41(3):317–25. doi: 10.1080/02813432.2023.2237074 (PMC10478594; doi:10.1080/02813432.2023.2237074)

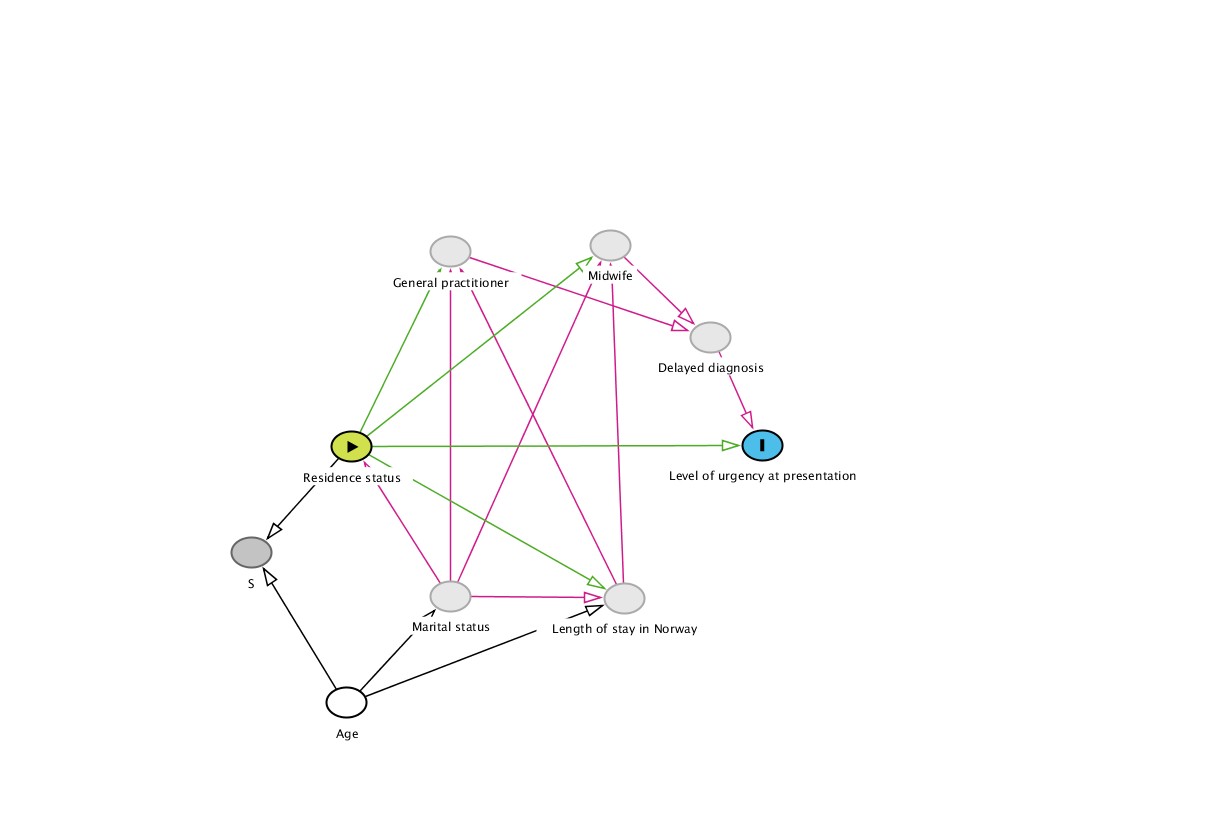

Supplement: Supplemental Material [file IPRI_A_2237074_SM3326.jpg]
